# Convolutional Neural Network for Depression and Schizophrenia Detection

**DOI:** 10.3390/diagnostics15030319

**Published:** 2025-01-30

**Authors:** Carlos H. Espino-Salinas, Huizilopoztli Luna-García, Alejandra Cepeda-Argüelles, Karina Trejo-Vázquez, Luis Alberto Flores-Chaires, Jaime Mercado Reyna, Carlos E. Galván-Tejada, Claudia Acra-Despradel, Klinge Orlando Villalba-Condori

**Affiliations:** 1 Laboratorio de Tecnologías Interactivas y Experiencia de Usuario, Unidad Académica de Ingeniería Eléctrica, Universidad Autónoma de Zacatecas, Zacatecas 98000, Mexico; carlosespino@uaz.edu.mx (C.H.E.-S.); luischaires@uaz.edu.mx (L.A.F.-C.); jaime.mercadoreyna@uaz.edu.mx (J.M.R.); 2 Centro de Investigación e Inovación Biomedica e Informática, Unidad Academica de Ingeniería Electrica, Zacatecas 98000, Mexico; acepeda@uaz.edu.mx (A.C.-A.); ktrejov@uaz.edu.mx (K.T.-V.); ericgalvan@uaz.edu.mx (C.E.G.-T.); 3 Vicerrectorado de Investigación, Universidad Nacional Pedro Henríquez Ureña, Santo Domingo 10203, Dominican Republic; c.acra@unphu.edu.do; 4 Vicerrectorado de Investigación, Universidad Católica de Santa María, Arequipa 04002, Peru

**Keywords:** depression, schizophrenia, motor activity, convolutional neural networks, diagnostic

## Abstract

**Background/Objectives**: This study presents a Convolutional Neural Network (CNN) approach for detecting depression and schizophrenia using motor activity patterns represented as images. Participants’ motor activity data were captured and transformed into visual representations, enabling advanced computer vision techniques for the classification of these mental disorders. The model’s performance was evaluated using a three-fold cross-validation, achieving an average accuracy of 95%, demonstrating the effectiveness of the proposed approach in accurately identifying mental health conditions. The objective of the study is to develop a model capable of identifying different mental disorders by processing motor data using CNN in order to provide a support tool to areas specialized in the diagnosis and efficient treatment of these psychological conditions. **Methods**: The methodology involved segmenting and transforming motor activity data into images, followed by a CNN training and testing phase on these visual representations. This innovative approach enables the identification of subtle motor behavior patterns, potentially indicative of specific mental states, without invasive interventions or self-reporting. **Results**: The results suggest that CNNs can capture discriminative features in motor activity to differentiate between individuals with depression, schizophrenia, and those without mental health diagnoses. **Conclusions**: These findings underscore the potential of computer vision and deep neural network techniques to contribute to early, non-invasive mental health disorder diagnosis, with significant implications for developing mental health support tools.

## 1. Introduction

Mental illnesses are a global issue that significantly impacts various aspects of human life. These disorders arise from genetic predispositions and environmental influences [1]. According to the agreed-upon definition, a mental disorder is characterized by clinically significant disturbance in an individual’s cognition, emotional regulation, or behavior, which causes significant distress or impairment of personal functioning [2]. According to the National Alliance on Mental Illness (NAMI), one in five adults may be diagnosed with a psychiatric disorder, making mental illness one of the main challenges in global health [3]. Among the most disabling and expensive disorders are major depressive disorder, schizophrenia, bipolar disorder, and autism [4]. These conditions affect people early on, significantly influencing their physical, emotional, and social development [1,5]. One of the most alarming aspects of mental illnesses is the decreased life expectancy of affected people, due both to medical complications and the increased risk of self-harming behavior or suicide. This panorama highlights the importance of addressing these diseases from a comprehensive approach, which includes prevention, early diagnosis, and access to interdisciplinary treatments [3,6].

**Figure 1:**
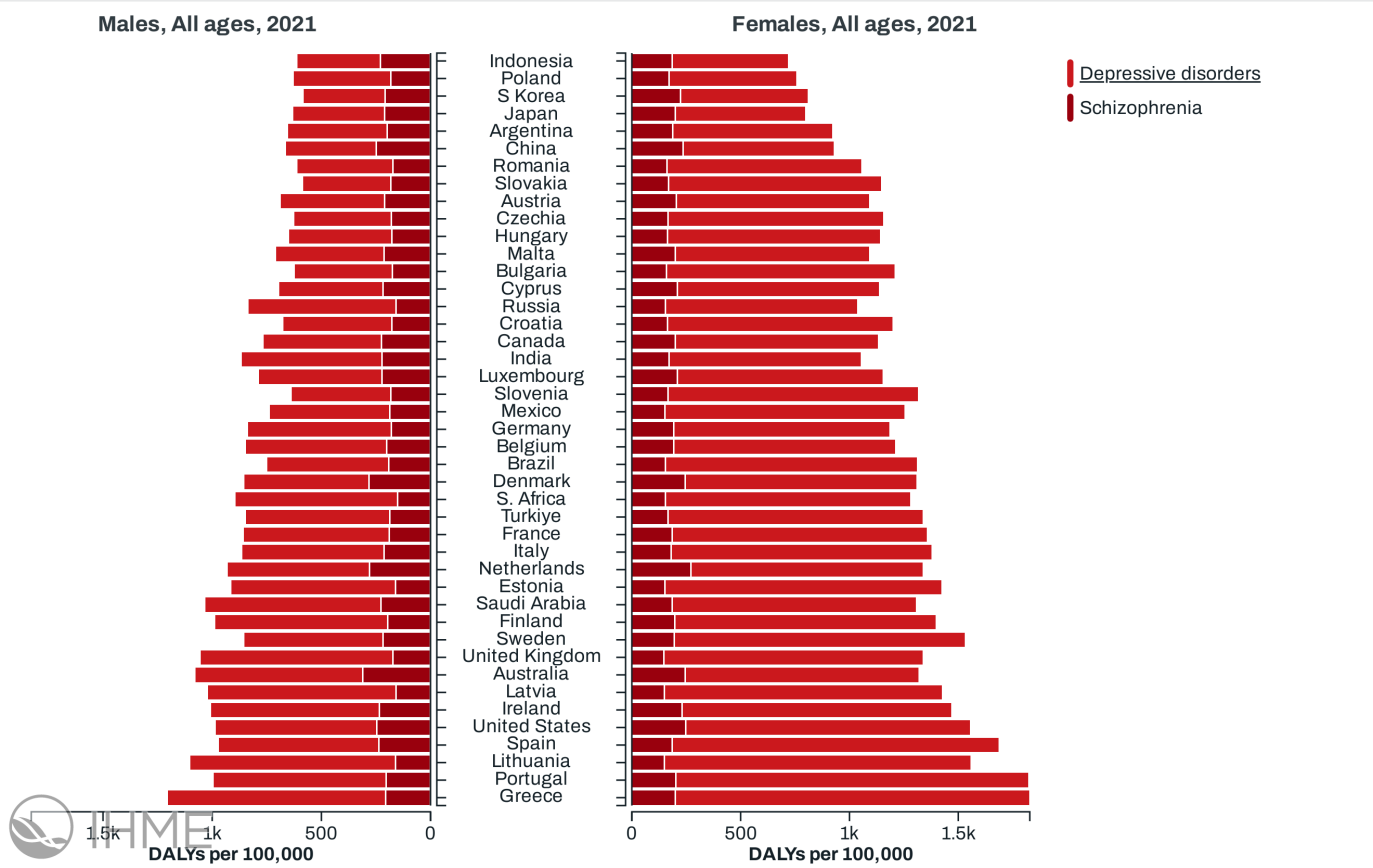
Global burden of depressive disorders and schizophrenia: Disability-Adjusted Life Years (DALYs) per 100,000 by gender in 2021 [7].

In 2021, psychiatric disorders such as schizophrenia and depressive disorders showed significant variations between countries and sexes, with notable differences in disease burden measured in Disability-Adjusted Life Years (DALYs) per 100,000 population, as shown in Figure 1. For example, in China, depressive disorders were much more prevalent in women (696.39 DALYs per 100,000) compared to men (415.94 DALYs per 100,000), indicating a higher burden for women in this region. Regarding schizophrenia, the rates were more similar between men and women, although slightly higher in men (for example, 248.65 DALYs per 100,000 in men compared to 235.43 in women in China). In countries such as Australia, depressive disorders had a particularly high burden, especially among women (1077.06 DALYs per 100,000), suggesting a higher prevalence of this condition compared to other regions. In contrast, in countries such as Angola, the rates for both disorders were significantly lower, with values of 96.96 DALYs per 100,000 in men for schizophrenia and 747.24 DALYs per 100,000 in men for depressive disorders, reflecting a lower burden compared to more developed nations. These data highlight the variability in the global burden of these mental disorders, with significant differences between genders and regions [7].

Major depressive disorder (MDD) is one of the most common mental illnesses; according to the World Health Organization (WHO), depression is a disorder that can affect anyone and represents a significant challenge to public health [8]. Its effects include reduced work capacity and reduced quality of life, and it constitutes a major risk factor for suicide and other adverse health outcomes [6,9]. According to the 11th revision (ICD-11) of the International Statical Classification of Diseases and Related Health Problems, to qualify for the diagnosis, an individual must exhibit five or more symptoms, one of which must be depressed mood or anhedonia [10]. The clinical symptoms of depression include a depressed mood, loss of interest, changes in weight or appetite, and increased probability of committing suicide [11]. The assessment of depression may serve a diagnostic purpose, aiming to determine the presence or absence of criteria established by DSM-5 or ICD-11. This process involves clinical interviews or the use of validated assessment instruments that have demonstrated robust psychometric properties to evaluate depression. Among the most used tools are Beck Depression Inventory II (BDI-II) [12], the Hamilton Depression Rating Scale [13], the Montgomery–Sberg Depression Rating Scale [14], the Patient Health Questionnaire-9 (PHQ-9) [15], the Zung Self-Rating Depression Scale [16], and the Center for Epidemiological Studies Depression Scale (CES-D) [17].

On the other hand, another of the most severe psychiatric disorders is schizophrenia; the term encompasses a set of disorders with heterogeneous etiologies and clinical manifestations, therapeutic response, evolutionary course, and prognosis, which can vary from patient to patient, and even within the same patient for the progression of the disorder [18,19]. It characteristically appears at the beginning of adulthood and generally accompanies the patient throughout their life, causing suffering to those who have it and their families, as well as a significant deterioration in quality of life [20]. People with schizotypal personality disorder share common phenomenological, genetic, biological, outcome, and response characteristics to treatment with more severely ill patients with chronic schizophrenia [18,20]. Schizophrenia is a complex psychiatric disorder whose assessment and diagnosis pose significant challenges due to the variability of symptoms and the lack of specific biological markers [21]. The cause of this neural disorder is unknown, but neuroscientists believe that the interaction between genes and several environmental factors may be the leading cause [22]. The diagnostic criteria, according to international classifications (ICD-11 and DSM-5), indicate the presence of delusions, hallucinations, language disturbances, psychomotor alterations, apathy, emotional incongruence, anhedonia, and social withdrawal. According to the DSM-5, if these symptoms resolve within less than 1 month, it is considered a brief psychotic disorder; if they persist for 1 to 6 months, the condition is termed schizophreniform disorder; and if they last for more than 6 months, the patient is diagnosed with schizophrenia [23].

Although complementary explorations exist that are sufficiently sensitive or specific in certain contexts, these are not yet sufficient to establish a definitive diagnosis. Therefore, the diagnosis of schizophrenia remains predominantly clinical and is based on identifying the criteria established by international classifications, such as the ICD-11 and the DSM-5 [24]. To complement the clinical diagnosis, diagnostic interviews and psychometric scales have been developed to more precisely describe the weight of various symptoms, as well as to assess their severity and the functional repercussions that the disorder generates in the individual’s life [24].

The use of these tools not only optimizes the diagnostic process but also facilitates the planning of personalized therapeutic interventions and the monitoring of outcomes over time, promoting an evidence-based and comprehensive approach to the management of schizophrenia.

Despite advances in knowledge about both disorders, current diagnostic and treatment approaches remain inadequate in many cases, underscoring the need to develop better predictive and personalized tools. The combination of genetic studies, neuroimaging, and advanced AI technologies offers a promising approach to improve diagnostic accuracy and treatment efficacy for depression and schizophrenia [25]. Analysis of large volumes of data through machine learning algorithms is also enabling deeper insights into underlying mechanisms, which could facilitate earlier and more targeted intervention.

### Related Work

In recent years, artificial intelligence (AI) has emerged as a key tool in the diagnosis and treatment of psychiatric disorders, offering new insights through the analysis of large volumes of genetic data, neuroimaging, and other biomarkers. This article presents a review of several recent studies on the use of AI in the early diagnosis and prediction of disorders such as schizophrenia, depression, and bipolar disorder, based on gene expression analysis, neuroimaging, clinical data, and motor activity.

Schizophrenia, a complex mental disorder, presents diagnostic difficulties due to its heterogeneous nature. Wagh et al. [26] conducted a systematic review of studies analyzing gene expression in peripheral blood as a potential biomarker for this disorder. The identification of genetic biomarkers in blood could offer a non-invasive approach to the diagnosis and monitoring of schizophrenia. Li et al. [27] reinforce this idea by identifying specific biomarkers through RNA sequencing, which offers a promising tool for early diagnosis of schizophrenia. These advances underscore the potential of AI to analyze large volumes of genetic data, facilitating the identification of patterns that might not be detected using traditional methods. Likewise, Zhu et al. [28] investigated the use of machine learning algorithms to diagnose schizophrenia based on gene expression in peripheral blood. The integration of AI in this context has been shown to improve diagnostic accuracy by combining genetic data with clinical features, which opens new possibilities for early diagnosis and early intervention in schizophrenia. Machine learning algorithms, such as neural network models, can analyze large databases to identify patterns and make accurate predictions about disease onset and progression.

On the other hand, the use of neuroimaging in combination with AI to improve the diagnosis of schizophrenia has been the subject of several investigations. De Filippis et al. [29] conducted a comprehensive review of studies employing AI to analyze structural and functional brain images. The use of machine learning techniques makes it possible to identify patterns in the images that are not perceptible to the naked eye, facilitating early diagnosis and assessment of schizophrenia progression. These multimodal approaches are essential to develop more accurate and personalized diagnostic tools.

Also, advances in monitoring technologies and data analysis techniques have facilitated the evolution of innovative methods for the diagnosis of mental illnesses. Depression, being one of the most prevalent psychiatric disorders, has been the subject of numerous studies [30,31,32,33,34,35,36,37,38,39] exploring alternative methods for its early detection, among which motor activity analysis stands out. This approach is based on the premise that depression can be reflected in altered motor activity patterns, allowing the identification of possible depressive episodes without the need for invasive interventions. In this context, there are several studies that have proposed innovative methodologies using technologies such as actygraphs and AI algorithms for the detection and classification of depression, opening up new possibilities in the field of mental health; as monitoring technologies and machine learning and deep learning algorithms evolve, such methodologies are likely to play a key role in improving methods for diagnosing and treating depression. In time, these approaches could be extended to other mental disorders, contributing to the creation of smarter, more personalized healthcare systems. That is why the present research work has as its main purpose to generate a model capable of identifying different mental disorders through the processing of motor data using convolutional neural networks in order to provide a support tool for the areas specialized in the diagnosis and treatment of these psychological conditions in an efficient way.

## 2. Materials and Methods

**Figure 2:**
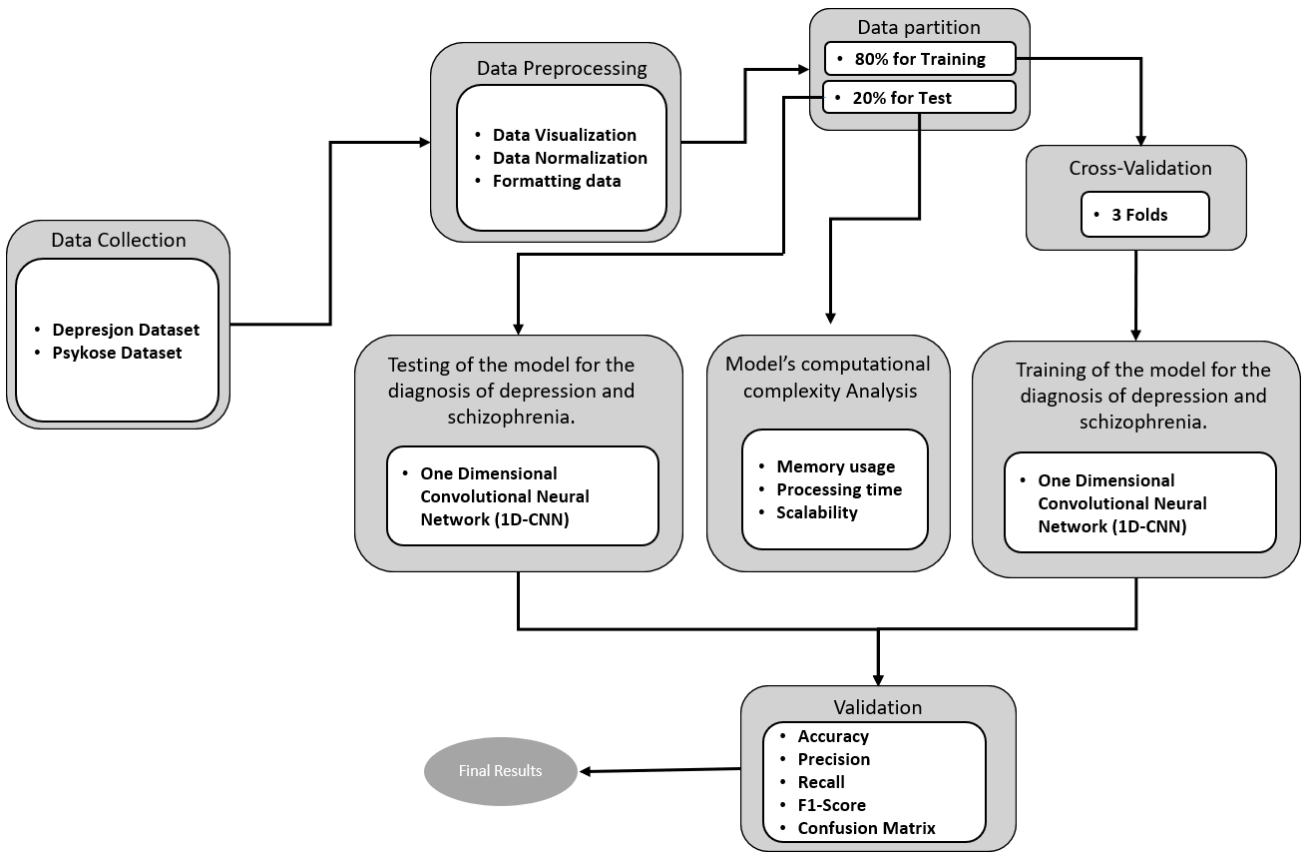
Proposed methodology for the development of an intelligent model for the preventive diagnosis of mental disorders using motor activity as a data source.

The methodology proposed to conduct the present study is summarized as shown in Figure 2. First, a detailed explanation of the process of collecting participant motor activity data and the source from which the information was obtained is given. Second, an analysis of the motor signal and a visual representation of each of the cases is performed in order to obtain an overview of the behavior of the data and to validate the possibility of generating models capable of making a timely diagnosis of depression and schizophrenia. Third, the acquired dataset is consolidated by grouping the 3 different cases—depression, schizophrenia, and control—to divide them into two segments (training and test) in order to create an intelligent model of preventive diagnosis of the mental illnesses addressed in this research. In the fourth stage, the first segment of data goes through a k-fold cross-validation process that generates a classification model that is then tested with the second segment of data known as blind testing. Finally, the model is validated with some of the most used metrics in the area of artificial intelligence in each of the fold and blind tests.

### 2.1. Data Collection

The first dataset, known as Depression: A Motor Activity Database of Depression Episodes in Unipolar and Bipolar Patients, presented by Ceja et al. in [40], contains sensorimotor activity data from patients with some degree of depression. In this dataset, 23 patients were diagnosed with unipolar and bipolar depression, 5 of the study subjects were hospitalized during the data collection period, and 18 were outpatients. A specialist physician assessed the level of severity of depression using the instrument known as the Montgomry–Asberg Depression Rating Scale (MADRS) at the beginning and end of the collected motor activity records. This dataset also contains motor data from 32 non-depressed patients known as the control group, which is composed of 23 hospital employees, 5 students, and 4 former patients without current psychiatric symptoms. The updated information and the dataset can be accessed through the following link: http://datasets.simula.no/depresjon/ accessed on 11 December 2024.

Table 1 depicts a cohort of young and middle-aged adults, presenting a high prevalence of affective disorders, predominantly unipolar depression. The absence of melancholia and the low frequency of hospitalization may suggest a relatively less severe clinical profile, both in terms of symptomatology and hospital treatment. In addition, most participants are unemployed or economically inactive, which may influence the observed disorders. Educational level is predominantly basic to intermediate level. These factors should be taken into account when interpreting the results and designing intervention or follow-up strategies.

The sample exhibits a balanced gender distribution, with a higher concentration of participants in the 20–34 years age range, which constitutes between 22.58% and 19.35% of the total sample across both genders. The average number of days spent varies from 12.00 to 14.00 days, with the 60–69 year and 50–54 year age groups showing slightly higher averages (14.00 and 12.83 days, respectively). The most prevalent age categories are 25–29, 30–34, 45–49, and 50–54 years, each representing between 16.13% and 19.35% of the sample. The gender-based distribution shows similar average days for both females and males, with values around 12.14 and 12.60 days, respectively. It is important to note that the Affect, Melancholy, Hospitalization, Education, Marriage, Work, MADRS1 (Initial), and MADRS2 (Final) columns are depopulated for the control group, as specific data on these factors were not available in the dataset provided as shown in Table 2.

On the other hand, the second dataset, known as PSYKOSE: A Motor Activity Database of Schizophrenia Patients, presented by Jakobsen et al. in [33], is composed of motor data collected from 22 psychotic patients hospitalized in the long-term psychiatric area of Haukeland University Hospital, consisting of 3 females and 19 males with a mean age of 46.2 ± 10.9 years.

This group consists of 3 women and 19 men, with an average age of 46.2 ± 10.9 years. According to the dataset, paranoid schizophrenia predominates (77.3%), which is consistent with studies showing this form as the most common subtype of schizophrenia. Most patients do not have migraines (81. 8%), but 18. 2% show co-existence. This could open a research line on comorbidities. Less than a third of the patients are treated with clozapine (31.8%), a medication commonly reserved for more severe or treatment-resistant cases. This demographic information can be useful for understanding the general characteristics of the patient group and guiding future analyses or research. It is pertinent to note that the demographic data presented in Table 3 correspond to the results obtained from the analyzed dataset.

Each of the study subjects was diagnosed with schizophrenia using a semi-structured interview based on the criteria of the Diagnostic and Statistical Manual of Mental Disorders IV (DSM-IV), which is a classification system for mental disorders that provides descriptions of diagnostic categories, in order for clinicians and researchers in the health sciences to diagnose, study, exchange information, and treat various disorders. The current edition is the fifth edition, known as DSM-5, and was published on 18 May 2013 [41].

In addition, the patients’ psychotic symptomatic status at the time was rated on the Brief Psychiatric Rating Scale (BPRS), which is often used to measure the general psychopathology of patients with schizophrenia. Briefly, BPRS consists of 18 items rated from 1 to 7, with higher total scores indicating a more severe condition [42].

The motor activity of the two datasets (Depresjon and Psykose) was collected using a right wrist actigraph, the Actiwatch, model AW4, manufactured by Cambridge Neurotechnology Ltd., Cambridge, UK.

In the same way as the first dataset mentioned at the beginning of this subsection, the PSYKOSE dataset takes the same motor data from the control or healthy patient group. The dataset can be found at the following link: https://datasets.simula.no/psykose/ accessed on 11 December 2024.

### 2.2. Data Preprocessing

The preprocessing stage consists of three processes that are key to the present study; the first is to visually represent the behavior of the motor activity signal, since data visualization is an indispensable part of the scientific process. Efficient visualization allows scientists to understand the data and give them a proper interpretation, and this process can be achieved with tools to specify a graph that provides a good balance between efficiency and flexibility. Within the scientific ecosystem of Python, there are libraries such as Seaborn that provide an interface to matplotlib that allows for exploring data and creating prototypes and visualizations, while retaining much of the flexibility and stability to produce high-quality graphs, allowing for visualizing a wide range of datasets that are represented in tabular form [43]. These functionalities allow for visualizing the motor activity of the different psychological conditions studied in this research.

On the other hand, it is also proposed to visually represent depression and schizophrenia through motor activity to improve the understanding and communication of these conditions, also facilitating the identification of patterns through deep learning algorithms such as convolutional neural networks (CNNs) that help in the diagnosis, detection, monitoring, and support to psychology professionals.

A Min–Max scaling technique was used to represent the different mental disorders through images. This procedure transforms the original motor activity values ($x_{i}^{\prime }$) into a defined range—in this case, between 0 and 255. The transformation was performed according to Equation (1).

(1) $$x_{i}^{\prime }=\mathrm{Min}+\frac{(x_{i}-\min\left(x\right))\cdot \left(\mathrm{Max}-\mathrm{Min}\right)}{\max(x)-\min(x)}$$

The second process in the preprocessing phase is to perform a normalization of the data; this is an essential step to achieve good classification performance before evaluating the data in deep learning algorithms. Data normalization involves the transformation of features into a common range so that larger numerical feature values cannot dominate smaller numerical feature values [44]. This process was carried out using *z* normalization or *z* score as shown in Equation (2).

(2) $$z_{i}=\frac{x_{i}-\bar{x}}{\sigma }$$

Finally, it is necessary to give the appropriate format to the dataset in order to process it. The process used was based on the research work entitled “Two-Dimensional Convolutional Neural Network for Depression Episodes Detection in Real Time Using Motor Activity Time Series of Depressions Dataset”, where a week was selected in one-minute intervals to generate a new dataset [30]. However, for this specific case, new observations related to the cases of participants with schizophrenia were added as shown in Equation (3). The output values represented as 0, 1, and 2 relate to the control, depression, and schizophrenia cases, respectively.

(3) $$A=\begin{array}{cccccccccc} \; & 00:00 & 00:01 & 00:02 & 00:03 & 00:04 & \; & 23:59 & Output\\ V_{1,1}^{T}= & [178 & 127 & 530 & 157 & 0 & \ldots & 208] & 0\\ V_{1,2}^{T}= & [138 & 17 & 560 & 0 & 23 & \ldots & 280] & 0\\ V_{1,3}^{T}= & [198 & 157 & 450 & 157 & 0 & \ldots & 305] & 1\\ V_{1,4}^{T}= & [248 & 157 & 120 & 457 & 521 & \ldots & 892] & 1\\ \; & \; & & \; & \ldots & \; & & \; & & \\ V_{55,5}^{T}= & [15 & 566 & 486 & 102 & 452 & \ldots & 302] & 0\\ V_{55,6}^{T}= & [34 & 145 & 456 & 578 & 234 & \ldots & 245] & 1\\ V_{55,7}^{T}= & [563 & 879 & 457 & 976 & 764 & \ldots & 753] & 2\\ V_{55,1}^{T}= & [763 & 466 & 1245 & 532 & 46 & \ldots & 533] & 2\\ \; & \; & & \; & \ldots & \; & & \; & & \\ V_{80,2}^{T}= & [78 & 753 & 73 & 235 & 365 & \ldots & 563] & 0\\ V_{80,3}^{T}= & [124 & 23 & 655 & 78 & 124 & \ldots & 76] & 1\\ V_{80,4}^{T}= & [567 & 564 & 23 & 134 & 987 & \ldots & 123] & 0\\ V_{80,5}^{T}= & [345 & 85 & 762 & 67 & 1543 & \ldots & 788] & 2 \end{array}$$

### 2.3. Training of Convolutional Neural Network

Once the data analysis, visualization, normalization, and formatting processes were completed, the CNN architecture was established, which was defined in the same way as that proposed by Espino-Salinas et al. [30], as shown in Table 4.

As shown in the table above, the input data also took dimensions of 30 × 48, which were calculated from the number of data captured per day (1440); the difference with the dataset presented in previous research is the significant increase in the number of observations and the incorporation of schizophrenia as a new group to be diagnosed by using motor activity as a data source.

CNNs are multilayer artificial neural networks whose main purpose is to identify, recognize, and classify objects, as well as detect and segment objects in images. These networks are frequently used in visual identification, medical image analysis, image segmentation, natural language processing, and now as a possible technique for the analysis of images represented through motor activity. CNNs can automatically identify key elements from input [45].

The CNN taken as a reference is made up of different elements. The first is the convolutional layer, which is basically a set of kernels or filters *L* that are applied to an input image *K* and are useful to extract features from the input data. A kernel learns to extract meaningful features, called feature maps, that accentuate relevant information from input data by adjusting randomly chosen initial values known as weights during the network training process [45]. This process is summarized as shown in Equation (4).

(4) (K−L+1)

Another relevant element used in 2D-CNN is the Rectified Linear Unit (ReLU) activation function. ReLU is one of the most commonly used activation functions for CNNs where each convolution layer is connected to this function. As shown in Equation (5), ReLU discards positive inputs to keep their values unchanged and generates zero for all negative inputs [46].

(5) $$f\left(x\right)=\left\{\begin{array}{cc} x & \mathrm{si}\;x\geq 0\\ 0 & \mathrm{si}\;x<0 \end{array}\right.$$

After each convolutional layer, a max-pooling procedure is performed that consists of a sliding window of a given size k×k that passes over the feature map and selects the maximum value of the elements within that region producing a new reduced feature map [47]. The max-pooling process for a feature map *X* can be expressed as shown in Equation (6).

(6) $$Y_{i,j}=\max_{\left(p,q)\in \mathrm{ventana}(i,j\right)}X_{p,q}$$

- $Y_{i,j}$ is the value of the reduced feature map at position (i,j).
- Window (*i*,*j*) represents the indices (p,q) corresponding to the region of the original feature map *X* covered by the window centered at (i,j).

Finally, the network is made up of four fully connected layers (FCLs) and an output layer of 3 perceptrons with a Softmax activation function, an essential component of deep learning models, used mainly in the last layer for multiclass classification [48]. The function converts the input values into probabilities, assigning each class a value between 0 and 1 through Equation (7), making sure that the sum of all probabilities is 1.

(7) $$\mathrm{softmax}\left(z_{i}\right)=\frac{e^{z_{i}}}{\sum_{j=1}^{K}e^{z_{j}}}$$

The selected parameters are adequate due to the size of the images, abd using a 3 × 3 kernel ensures spatial features are extracted and patterns such as edges, textures, and shapes in small images such as those used in this study are detected. On the other hand, a reduced number of 4 convolution layers ensures a more optimized training process by reducing the time and processing power to generate the intelligent diagnostic model of the different cases. Likewise, adding 3 max-pooling layers reduces the spatial dimensionality of the features, decreasing the size of the processed images, improving generalization, and retaining the most important information of the features. We also added 5 dropout layers that randomly turn off a percentage of neurons during training to prevent overfitting. Finally, it converts the two-dimensional data resulting from the convolutional layers into a one-dimensional vector and prepares the extracted features to be processed by the fully connected dense layers to classify the motor data of different mental states.

For the CNN benchmark training process, the data were divided into a training set (70%) and a test set (30%). The training set underwent a k-fold cross-validation process with k=3. Meanwhile, the test set was used for blind testing and computational complexity analysis, as the model is designed to perform “real-time” inferences, detecting depression and schizophrenia by collecting data throughout the day.

### 2.4. Validation

One of the most important phases of this research is to quantify the performance of the deep learning model through CNN to detect depressive, schizophrenic, and control states; this is achieved using validation metrics. A proper validation tests the robustness of the model to any possible input variation, and also validation provides an unbiased estimate of model performance in the face of never before seen data similar to those used during training [49].

For classification tasks, one of the most viable options is confusion matrices that can summarize the results in a way that shows graphically how many times one true class is predicted to be another. The confusion matrix can reveal which classes are commonly confused [49].

**Figure 3:**
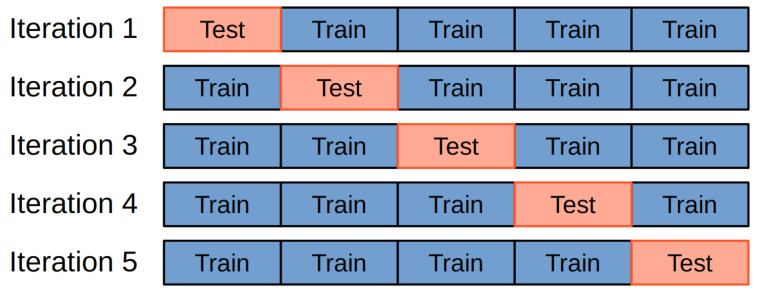
Example of *k*-fold cross validation process for a *k* = 5.

Cross-validation is also one of the most commonly used metrics in machine learning and deep learning used to evaluate a classification model. This method includes k-fold cross-validation, which aims to select training and test data sets to create models with higher accuracy, as shown in Figure 3. Another goal of k-fold cross-validation is to avoid overfitting in the model training process. [50].

Additionally, four validation metrics were considered to evaluate the model’s performance in detecting different mental disorders: *Accuracy* (Equation (8)), *Recall* (Equation (9)), *Precision* (Equation (10)), and *F*1-Score (Equation (11)).

(8) $$Accuracy=\frac{TP+TN}{TP+TN+FP+FN}$$

(9) $$Recall=\frac{TP}{TP+FN}$$

(10) $$Precision=\frac{TP}{TP+FP}$$

(11) $$F1=2\cdot \frac{Precision\cdot Recall}{Precision+Recall}$$

*TP*, *TN*, *FP*, and *FN* refer to True Positive, True Negative, False Positive, and False Negative, respectively, for each class in a multi-class classification problem.

## 3. Results

**Figure 4:**
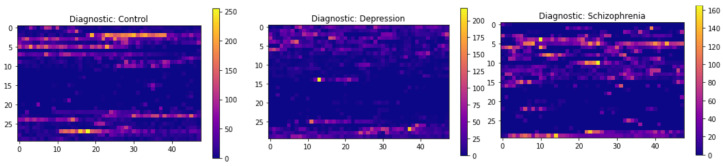
Images of the different diagnoses: control, depression, and schizophrenia represented through motor activity.

This section presents the results obtained from the experimentation carried out. First, a visual representation (images) of the different mental disorders studied in this research, such as depression and schizophrenia, was made for motor activity, as shown in Figure 4. This type of representation, besides allowing an understanding of these mental disorders, also helps to identify the changes between one disorder and another, which helps to establish a first approximation to detection through CNNs.

As can be seen in the image above, there are certain identifiable patterns that can provide important information about each mental disorder analyzed in this study. The most notable case is the image related to depression, where it can be seen that there is less activity compared to schizophrenia and control cases—this is highlighted by the contrasts in the image (ranges close to 150 represent high motor activity of the participants and those close to 0 null or little motor activity). On the other hand, schizophrenia shows very high levels of motor activity, even higher than control cases, which demonstrates the feasibility of implementing CNNs to detect this type of psychological condition by representing motor activity through images, since CNNs have demonstrated performance in several tasks such as computer vision [51].

**Figure 5:**
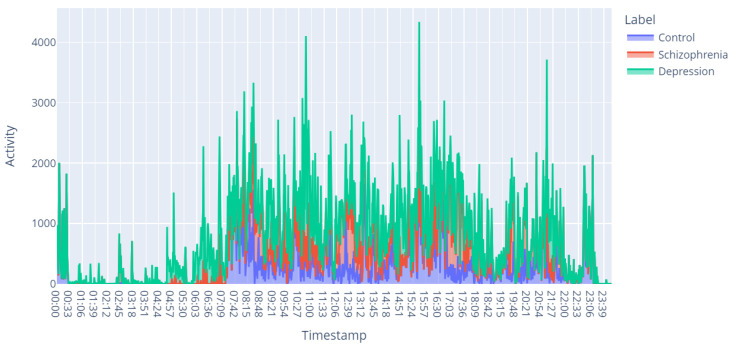
Example of samples collected with Actiwatch during 24 h. for different mental disorders.

Another useful perspective to identify possible changes in the behavior of study subjects is through the visualization of the motor activity signal over the span of a day as shown in Figure 5. This approach also makes it possible to establish a starting point that justifies this type of data as a source of information for the detection of mental disorders.

**Figure 6:**
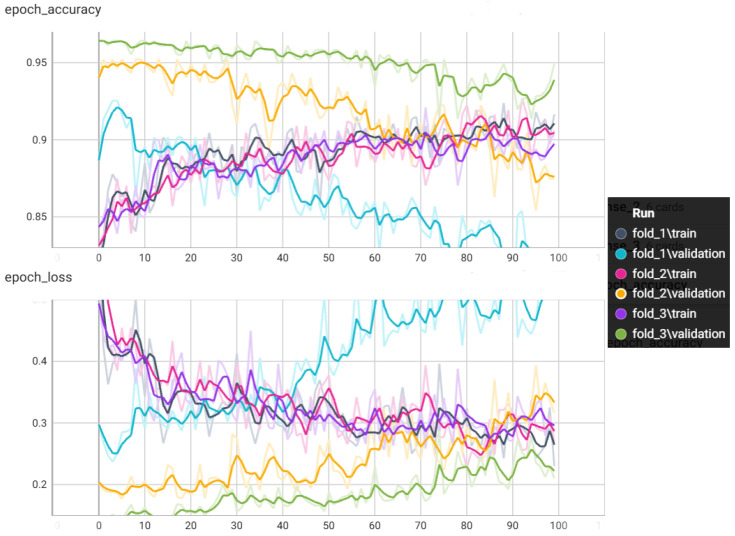
Loss and accuracy curves on training data using cross validation with k=3 for 100 epochs.

Taking into account the previous analysis, in the 2D-CNN training phase, 1436 motor activity images were used; 70% of the images, equivalent to 1005 images (492-Control, 262-Depression, and 251-Schizophrenia), were used to train the network through a K cross-validation process with k=3 during 100 epochs. Figure 6 shows the accuracy and loss curves during this process for each of the folds.

Pathar et al. [52] state that for a model to have acceptable validation, during CNN training, the validation loss should be similar to the training loss. In case the validation loss is less than the training loss, this means that the model is underfitted and should be trained with a higher number of epochs. For the case of the CNN model trained for schizophrenia and depression detection, the above condition was fulfilled during fold 2 and 3, since the validation and training loss followed the same similar pattern at the end of the epochs.

The resulting training model performed quite acceptably for each of the folds as shown in Table 5. These results demonstrate the feasibility of detecting depression as well as schizophrenia.

It should be noted that despite the good results obtained in the training phase, it is necessary to perform additional tests to check the model’s ability to generalize the input data, i.e., how well or how poorly the model performs with data that it does not know but that are similar to the data used during training. For this, we took the remaining 30% of the data (222-Control, 104-Depression, and 105-Schizophrenia) that was not used for training the model, obtaining the results presented in Table 6.

Although the overall performance obtained with an accuracy of 84% is lower compared to the results obtained during the validation of the model in the training phase, they are still quite acceptable results that are even comparable with previous works.

**Figure 7:**
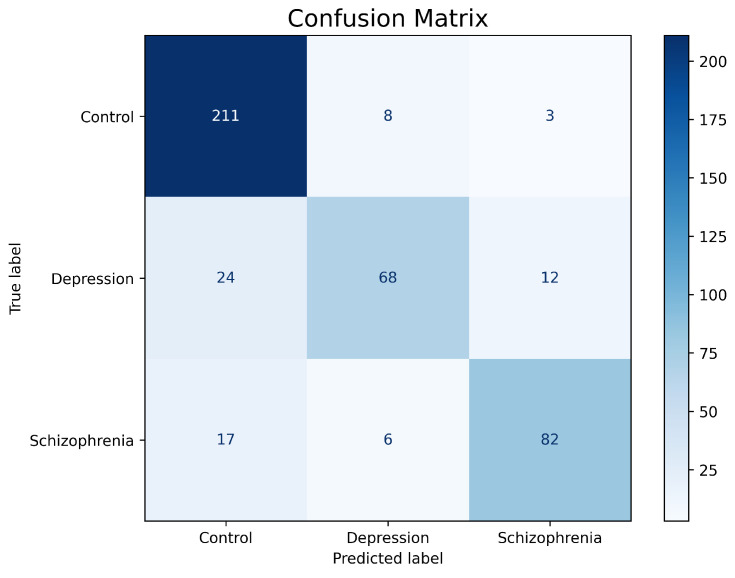
Confusion matrix resulting during blind testing of the model for the detection of depression and schizophrenia.

The results presented above also show that the proposed model had similar accuracy, recall, and F1-score for each of the diagnoses or cases. However, given the imbalance of classes, where the control cases were slightly higher than those of depression and schizophrenia, the model was more susceptible to detect a higher number of control classes as shown in the confusion matrix presented in Figure 7.

The resulting confusion matrix represents that of the 222 control subjects, the 104 subjects with depression, and the 105 subjects with schizophrenia; the model managed to classify 211, 68, and 82, respectively, correctly with an inference time of 0.321 s with a memory usage of 688.17 MB.

In summary, the findings obtained highlight the performance of the convolutional neural network in the detection of depression and schizophrenia, evidencing both strengths and areas for improvement in the evaluated metrics. These results provide a solid basis for interpreting the factors that influence the model’s performance and its applicability in clinical contexts. Next, the implications of these findings will be analyzed, contrasting them with previous research and exploring their practical relevance and possible limitations.

## 4. Discussion

The results of this study highlight the importance of motor activity as a biomarker of mental health disorders, and offer a novel and non-invasive approach for their detection.

Although there are interesting proposals that also use machine learning tools for the detection of mental illness, some, such as Zhu et al. [28], who specifically detected schizophrenia, are invasive, since the data used to train and test the models are extracted from biological samples—in this case, peripheral blood—which also require complicated processing by requiring specialized material and equipment that ultimately increases the complexity of the data collection stage.

Transforming motor activity data into visual representations allowed the convolutional neural network (CNN) to effectively analyze and classify patterns that might otherwise remain imperceptible to traditional diagnostic methods. This highlights the advantage of deep learning models to identify complex and subtle features within high-dimensional data compared to previous work, where Rodríguez-Ruiz et al. [53] performed a study related to the detection of schizophrenia and depression using motor activity as a source of information and random forest as a machine learning algorithm to classify these types of conditions. However, in their proposal, the random forest algorithm focuses on manually extracted statistical features and works well for these well-structured and tabular data. CNNs are superior in processing a visual representation of motor activity data by automatically extracting complex patterns by collecting all the necessary information during a day. In another alternative, Galvan-Tejada et al. [34] proposed obtaining temporal and frequency statistical features from the depressive database to classify healthy people and people with depressive states, which are then subjected to a feature selection step using the genetic algorithm (GA) “Greyhound”; although the machine learning model used (Random Forest) for classification is simpler in terms of hyper parameters compared to a CNN, the value of accuracy in the final classification of 64.03% is lower than that obtained in our experiment, which was 84%. It is also worth mentioning that the dimensionality in the final classification is different since their work only classifies people with depression and healthy people. It should be noted that their work reflects the association between a patient’s recorded daily activity and his or her depressive state.

On the other hand, proposals such as those of Espino-Salinas et al. [54] suggest that the use of a few time intervals is sufficient to establish a preventive diagnosis of depression, but the use of motor data from an entire day gives a more complete and robust view of individual patient behavior, allowing the collection of much more complex patterns, temporal trends, and behavioral variations associated with disorders such as schizophrenia and depression. While working with time interval data may be more efficient and practical in some scenarios, there is a risk of losing critical information. In addition, the high accuracy achieved underlines the reliability of this approach, suggesting its potential for integration into real-world clinical settings or wearable technology systems for continuous monitoring.

Also, Zakariah et al. [35] explored an integration of Uniform Manifold Approximation and Projection (UMAP) and neural networks along with other classification algorithms, showing how dimensionality reduction allows effective representation of motor activity data and enabled a high accuracy of 99.1% for diagnosing depression by combining multiple methodologies. However, the proposed CNN-based approach offers a significant advantage in both detecting schizophrenia and simplifying the workflow by avoiding the need for additional techniques such as UMAP, which could facilitate its implementation in practical applications.

Finally, the study by Price et al. [55] has an exploratory character and highlights the potential of unsupervised methods to discover natural clusters in motor data. The present study moves towards practical implementation by using CNNs to classify schizophrenia and depression with high accuracy. Despite this, both approaches suggest a complement to each other, where the proposed model provides an accurate diagnosis and the methods of Price et al. [55] offer valuable contextual information for understanding the data. Similarly, another work that aims to detect people with depression is that of Frogner et al. [38], who use a one-dimensional CNN that, when compared with a two-dimensional CNN, requires less computational resources for training and testing than the model explored by Shahid et al. [56]. Now, taking into account that the proposal of Frogner et al. [38] takes as its source the Depresjon database, their results in the testing stage of the F1-score metric for detecting only people with depression are slightly lower (0.64) than the values obtained by our model (0.73), indicating that, although the complexity of transforming unidimensional data to two-dimensional is higher, the results obtained at the end validate such transformation.

Another important aspect to discuss is the problem of class imbalance. In this study, a dataset with 1436 samples distributed in three classes was observed: “Control” with 697 samples (48.5%), “Depression” with 371 samples (25.8%), and “Schizophrenia” with 368 samples (25.6%). This imbalance suggests that the model may be biased toward the majority class during training, resulting in less accurate predictions for the minority classes.

The class imbalance observed in the original data directly impacts the recall of the “Depression” class, which can be critical in practical scenarios where false negatives have serious consequences, such as in clinical applications. This phenomenon is evidenced by the model not correctly identifying all cases of depression, possibly due to the lower representation of this class in the dataset.

However, the excellent performance in the class “Schizophrenia” could be influenced by its higher representation, which allows the model to learn more generalizable patterns for this class. However, it is important to note that the high accuracy in this class could be benefited by the lower risk of false positives compared to the minority classes.

Further studies aim to solve such problems through techniques such as Synthetic Minority Oversampling (SMOTE) that allow for increasing the number of samples in these classes by generating new synthetic data [57]. An additional technique proposed to be applied is Adaptive Synthetic (ADASYN), which consists of using a weighted distribution for different examples of minority classes, according to their level of difficulty in learning, where more synthetic data are generated for the examples of minority classes that are easier to learn [58].

Despite this, the model shows a solid overall performance, with an accuracy of 95% and a high F1-score in all three categories (schizophrenia, depression, and control). These metrics indicate that the system can be a useful adjunct in the early detection of mental conditions, reducing the exclusive reliance on traditional methods, such as clinical interviews and questionnaires, which tend to be more subjective and time-consuming. It is also worth noting that the compact and efficient design of models such as the proposed one, which employs a relatively simple but effective architecture, makes it ideal for implementation on portable devices.

## 5. Conclusions

This study underscores the substantial potential of Convolutional Neural Networks (CNNs) as an advanced diagnostic tool for mental health disorders, particularly depression and schizophrenia, through the analysis of motor activity represented visually. In comparison to traditional diagnostic methods that rely on manually extracted features, CNNs offer a distinct advantage. They have the capacity to autonomously learn and extract complex, non-linear patterns directly from images, eliminating the need for human intervention in feature selection. This capability makes CNNs more flexible and precise, enabling more accurate and reliable classification of mental health disorders. This automated process facilitates earlier detection, which is crucial for implementing timely and effective interventions.

The results of the study reveal that visualizing motor activity allows one to identify distinct patterns between various mental health conditions. For example, depression, often characterized by reduced motor activity, presents a stark contrast to schizophrenia, which typically involves heightened motor activity. By leveraging the power of CNNs, these subtle differences can be captured more effectively than with traditional methods. This is particularly relevant in the context of mental health diagnostics, as capturing such nuances in behavior can significantly enhance the accuracy of diagnosis, allowing for more tailored treatment options.

Despite the promising results, several limitations must be addressed in future research. First, while the model performed well during the training and validation phases, there is a need to enhance its generalization ability—its performance when exposed to unseen data. The real-world variability of data, such as individual differences in behavior and the presence of additional comorbidities, could affect the model’s detection accuracy. Therefore, further testing is required in diverse clinical contexts and with a wider range of patient populations to assess how robust the model truly is. Additionally, it is important to test the model across various settings, including outpatient and remote care environments, to determine its versatility and potential for widespread adoption.

Another challenge identified in this study is the class imbalance in the dataset. The number of control subjects exceeded those with depression or schizophrenia, which may have led to a bias in the model’s predictions. While the model still achieved a commendable accuracy rate, its tendency to classify control cases more accurately suggests that strategies to mitigate class imbalance are needed. Techniques such as oversampling the minority class or using more advanced algorithms designed to handle imbalanced datasets should be considered in future iterations to ensure that the model can classify all conditions more equally. By addressing this issue, the model could achieve more balanced and fair classification, leading to improved diagnostic outcomes.

Furthermore, one of the key advantages of this study is the use of a full day’s worth of motor activity data for analysis. This approach allows the model to capture a wide array of temporal patterns and behavioral variations throughout the day, which enhances the depth and accuracy of the analysis. However, this approach could be further strengthened by integrating the model with continuous monitoring technologies, such as wearable devices or sensors that track motor activity in real time. This would allow for dynamic, ongoing data collection, providing an even more comprehensive understanding of a patient’s motor behavior over time. Continuous monitoring would also enable real-time interventions, enhancing the responsiveness and personalization of mental health care. By collecting data continuously, this method could identify early signs of mental health deterioration, facilitating timely interventions and reducing the risk of exacerbating symptoms.

Incorporating continuous monitoring technologies also has the potential to transform the diagnostic process. Real-time data collection could not only provide a clearer picture of a patient’s condition but also enable interventions that are better suited to the patient’s specific needs. For instance, patients exhibiting early signs of depressive or schizophrenic episodes could receive intervention at the most crucial point, potentially preventing the onset of more severe symptoms. The ability to track a patient’s progress continuously through wearable devices would also allow clinicians to adjust treatment plans based on real-time feedback, ensuring that interventions are always aligned with the patient’s current condition.

Although the results obtained so far are very promising, the study highlights the need for further research to improve certain aspects of the model, particularly in terms of its ability to generalize to unseen data. In this study, while an acceptable performance was achieved, the inherent variability of real-world data and the complexity of mental disorders could affect the model’s performance in more diverse scenarios or with different patient populations. Generalization is key because a model that cannot adapt to new situations may not be reliable in real clinical contexts. Therefore, continued testing and adjustment of these models in different environments, with varied patient groups and conditions, is essential to ensure robustness and effectiveness in clinical practice.

Additionally, this study suggests that integrating CNNs with continuous monitoring technologies could take this approach to the next level. Wearable devices, such as motion sensors or other monitoring technologies, allow for the continuous collection of real-time motor activity data, facilitating more dynamic and accurate tracking of patients. This integration would not only enable the model to detect behavioral patterns at specific moments, but also to monitor the evolution of these patterns over time. By doing so, early signs of changes in motor activity that precede clinical symptoms could be identified, leading to faster and more personalized interventions. This type of continuous monitoring has the potential to significantly improve patients’ quality of life by detecting issues before they worsen, enabling immediate adjustments to treatment plans.

In conclusion, although the results obtained so far suggest that CNNs can be an effective tool in clinical practice and wearable devices offer enormous potential to transform the diagnosis and monitoring of mental conditions such as schizophrenia and depression. However, to ensure their success, challenges related to class imbalance, clinical validation, privacy, and acceptance by healthcare professionals need to be addressed. With further improvements and appropriate validations, this approach could become an essential component in mental healthcare, promoting more accurate diagnoses, early interventions, and continuous monitoring in everyday settings.

## Figures and Tables

**Table 1:** Demographic profile of the condition group.

Category	Option	Count	Percentage
Gender	Female	10	47.62%
	Male	11	52.38%
Age Group	20–24	1	4.55%
	25–29	2	9.09%
	30–34	4	18.18%
	35–39	5	22.73%
	40–44	4	18.18%
	45–49	4	18.18%
	50–54	3	13.64%
	55–59	1	4.55%
	60–64	1	4.55%
	65–69	1	4.55%
Aftype	Bipolar II (1)	6	28.57%
	Unipolar Depression (2)	14	66.67%
	Bipolar I (3)	2	9.52%
Melancholia	With Melancholia (1)	2	9.52%
	Without Melancholia (2)	19	90.48%
Inpatient	Inpatient (1)	2	9.52%
	Outpatient (2)	19	90.48%
Marriage	Marital Cohabiting (1)	9	42.86%
	Single (2)	12	57.14%
Work	Working/Studying (1)	9	42.86%
	Unemployed/Sick Leave/Pension (2)	12	57.14%
MADRS (Initial)	14–18	3	14.29%
	19–23	7	33.33%
	24–28	8	38.10%
	29+	4	19.05%
MADRS (Final)	13–18	5	23.81%
	19–23	6	28.57%
	24–28	7	33.33%
	29+	4	19.05%
Education (Years)	06–Oct	10	47.62%
	15–Nov	7	33.33%
	16–20	4	19.05%

**Table 2:** Demographic profile of the control group.

Category	Option	Count	Percentage
Control Group	Female	18	56.25%
	Male	14	43.75%
Age Group	20–24	7	22.58%
	25–29	5	16.13%
	30–34	6	19.35%
	35–39	4	12.90%
	40–44	2	6.45%
	45–49	5	16.13%
	50–54	6	19.35%
	60–64	1	3.23%
	65–69	1	3.23%

**Table 3:** Distribution of the sample according to gender, age, type of condition, presence of migraines, and prescribed medications.

Category	Category Description	Number	(%)
Gender	Male	19	86.4%
	Female	3	13.6%
Age	25–29	1	4.5%
	30–34	2	9.1%
	35–39	4	18.2%
	40–44	5	22.7%
	45–49	2	9.1%
	50–54	3	13.6%
	55–59	4	18.2%
	65–69	1	4.5%
Type	Paranoide	17	77.3%
	No paranoide	5	22.7%
Presence of migraines	Migraine-free	18	81.8%
	With migraines	4	18.2%
Medications prescribed	With Clozapine	7	31.8%
	Without Clozapine	15	68.2%

**Table 4:** Two-Dimensional Convolutional Neural Network (2D-CNN) architecture utilized in this study comprises layers such as convolutional (Conv), rectified linear unit (ReLU), max-pooling (Pool), and fully connected layers (FCLs).

Layer Type	Number of Filters	Size of Feature Map	Size of Kernel	Number of Stride	Number of Padding
Image Input Layer	30 (Height) × 48 (Width)				
Conv2d_1	48	26 × 44 × 48	3 × 3	1 × 1	1 × 1
ReLU 1	1	26 × 44 × 48			
Pool 1	48	13 × 22 × 48	3 × 3	2 × 2	1 × 1
Conv2d 2	48	11 × 20 × 48	3 × 3	1 × 1	1 × 1
ReLU 2	1	11 × 20 × 48			
Pool 2	48	5 × 10 × 48	3 × 3	2 × 2	1 × 1
Conv2d 3	48	3 × 8 × 48	3 × 3	1 × 1	1 × 1
ReLU 3	1	3 × 8 × 48			
Pool 3	48	1 × 4 × 48	3 × 3	2 × 2	1 × 1
1st FCL		900 × 1			
ReLU 4		900 × 1			
Pool 4		900 × 1			
2nd FCL		300 × 1			
ReLU 5		300 × 1			
Pool 5		300 × 1			
3rd FCL		100 × 1			
ReLU 6		100 × 1			
Pool 6		100 × 1			
4th FCL		2 × 1			
Sigmoid		2 × 1			
Output Layer		2 × 1			

**Table 5:** Results obtained in each of the 3 folds for training a model for detecting mental disorders.

Fold	Diagnostic	*Precision*	*Recall*	*F*1-Score
	Control	0.83	0.94	0.88
1	Depression	0.90	0.55	0.68
	Schizophrenia	0.77	0.88	0.82
*Accuracy*				0.82
2	Control	0.83	0.97	0.90
	Depression	0.92	0.80	0.86
	Schizophrenia	0.93	0.77	0.85
*Accuracy*				0.87
3	Control	0.96	0.99	0.96
	Depression	1.00	0.87	0.93
	Schizophrenia	0.94	0.92	0.93
*Accuracy*				0.95

**Table 6:** Results obtained during the blind test of the model for detecting mental disorders.

Fold	Diagnostic	*Precision*	*Recall*	*F*1-Score
	Control	0.84	0.95	0.89
Blind Test	Depression	0.83	0.65	0.73
	Schizophrenia	0.85	0.78	0.81
*Accuracy*				0.84

## Data Availability

The datasets can be accessed via the following link: http://datasets.simula.no/ and https://datasets.simula.no/psykose/ accessed on 14 December 2024.
